# What statistical methods are more appropriate for predicting recruitment at the design stage of a randomised controlled trial?

**DOI:** 10.1186/s13063-026-09900-3

**Published:** 2026-07-17

**Authors:** Freya J. Marks, Stephen J. Walters, Laura Sutton, Richard M. Jacques

**Affiliations:** Division of Population Health, School of Medicine and Population Health, The University of Sheffield, Barber House, 387 Glossop Road, Sheffield, S10 2HQ UK

**Keywords:** Recruitment prediction, Statistical methods, Randomised controlled trial

## Abstract

**Background:**

Effective prediction and monitoring of recruitment in randomised controlled trials (RCTs) is critical to ensuring the target sample size is met, with 37% of trials failing to do so. This research aimed to identify various statistical methods for predicting recruitment during the design stage of an RCT and implement them using case-study trials, with a view to determining which method is more appropriate.

**Methods:**

Various deterministic, Poisson and Bayesian methods were identified and applied to data from six previously conducted RCTs, with varying numbers of participants recruited, sites opened, durations and recruitment outcomes.

**Results:**

Poisson methods were found to be more appropriate for obtaining predictions at the design stage of a trial. They produced confidence intervals to model uncertainty, unlike deterministic methods, and exhibited narrower intervals than Bayesian methods using informative priors. For single-centre trials, a homogeneous Poisson process was recommended, whilst a Non-homogeneous Poisson Process could be more suitable for multicentre trials. However, the non-homogeneous approach yielded more conservative estimates and required site-specific start dates, which may be less readily available at the design stage. Limitations included a lack of usable software for method implementation, difficulties with parameter elicitation, and a lack of additional data required to implement more complex methods, all of which may hinder the application of statistical methods for predicting recruitment which occurs in only 10% of RCTs.

**Conclusions:**

To enhance transparency and reproducibility, it is recommended that RCTs publish the methods and parameters used in recruitment predictions. This may improve their accuracy, thereby reducing the cost and minimising the research waste associated with insufficient recruitment. Further research across a wider range of trials and methods may also be required due to the complexity of factors which influence recruitment and the variability in the accuracy of statistical methods across trials.

## Background

Failure to recruit to the target sample size in randomised controlled trials (RCTs) is the leading cause of trial discontinuation [1]. This can compromise both internal and external validity and generate research waste, where patients are unnecessarily exposed to or withheld from treatments with no valuable information acquired from the trial [2]. During the 2022–2023 period, the National Institutes of Health (NIH) financed $49 billion for medical research [3], and the National Institute for Health and Care Research (NIHR) granted £450 million to research projects, the majority of which were RCTs [4]. This highlights the critical importance of achieving recruitment targets to prevent the need for additional time and financial extensions.

The most recent review of recruitment in RCTs found that 37% of 379 trials published in the NIHR Journals Library between 1997 and 2020 did not recruit to their target sample size, with 20% needing to revise their target downwards [5]. Previous studies reported higher failure rates, with 69% of 114 trials funded by the UK Medical Research Council (MRC) [6] and 44% of 151 trials funded by the UK NIHR HTA Programme [7] not meeting their recruitment targets. A review of 133 trials published in six major journals: Annals of Internal Medicine, Annals of Surgery, British Medical Journal (BMJ), The Journal of the American Medical Association (JAMA), The Lancet and The New England Journal of Medicine (NEJM) between July and December 2004 found that, of the 84% which reported a target sample size, 21% failed to recruit to this number [8]. These reviews acknowledge substantial variability in recruitment amongst RCTs, noting that the complexities of recruitment failure stem from factors including participant age, deprivation, immigration status, health status, other commitments, attitudes towards research or healthcare, randomisation concerns, recruitment method and trial characteristics [9].

Given the costs and risks associated with poor recruitment, it would be beneficial to obtain accurate predictions of the timeframe required to achieve the target sample size. However, predictions made at the design stage of an RCT often underestimate the required timeframe, and there is a lack of consistency in the methods used for prediction. A survey of 69 statisticians within UK Clinical Research Collaboration (UKCRC)-registered clinical trials units and the European Clinical Research Infrastructure Network revealed that 10% used a statistical model, and 57% had a statistician leading recruitment prediction, though 81% believed they should have [10].

Recognising the significance of recruiting an adequate number of participants, the aim of this study was to identify and describe statistical methods for predicting recruitment to RCTs at the design stage and apply them to case-studies to assess their suitability.

## Methods

### Statistical methods for predicting recruitment

A literature search informed by previous reviews by Barnard et al. [11] and Gkioni et al. [12] identified five methods across three model classes that can be used to predict recruitment at the design stage of an RCT: deterministic unconditional, deterministic conditional, unconditional Poisson, conditional Poisson and Bayesian.

#### Deterministic unconditional

The deterministic, unconditional method estimates the recruitment period by dividing the target sample size by an expected linear recruitment rate, which is assumed to be fixed and is often estimated using previous trends at each research site [13]. A limitation, especially in multicentre trials, is the lack of consideration of individual site-specific rate variations and overall rate variation due to variable recruitment start dates. The assumption of a constant rate over time could lead to its overestimation, especially in the initial months of recruitment. A first-order recruitment model (FORM) for this method was presented by Comfort [14], with the following assumptions:

1 $${N}_{m}=\left({S}_{m}R {t}_{e}\right)+{N}_{0}$$

where

- ${N}_{m}$ is the total number of participants required
- ${S}_{m}$ is the total number of sites planned for recruitment
- R is the recruitment rate (number of participants per site per month)
- ${t}_{e}$ is the total duration of recruitment
- ${N}_{0}$ is the number of participants initially enrolled, if required

Depending on the requirements of the funder, this equation can be rearranged to give the enrolment duration as follows:

2 $${t}_{e}=\frac{{N}_{m}-{N}_{0}}{{S}_{m}R}$$

#### Deterministic conditional

To address limitations of the unconditional approach, a conditional approach incorporates prior information such as individual site recruitment rates and active recruitment periods [13]. This could be useful for determining the effect of including a site by delaying its recruitment start date. However, it remains limited by its fixed recruitment rate over time. Comfort expanded this method, introducing a second-order recruitment model (SORM) [14] whereby the second-order recruitment rate whilst sites are starting up, ${N}_{q}$, is given by

3 $${N}_{q}\left(t\le {t}_{s}\right)=\left(\frac{1}{2}{l}_{r}{t}^{2}+{S}_{0}t\right)R+{N}_{0}$$

and the linear recruitment rate after all sites have started recruiting is given by

4 $${N}_{l}\left(t>{t}_{s}\right)={S}_{m}Rt+{N}_{q}$$

where

- $$\begin{array}{c}{l}_{r}=\frac{\left({S}_{m}-{S}_{0}\right)}{{t}_{s}} \end{array}$$ is the site initiation rate
- ${t}_{s}$ is the average site start up time
- ${S}_{0}$ is the number of initial sites ready to recruit

The total study enrolment period, ${t}_{e}$, is the positive root solution of the following equation:

5 $${t}_{e}=\frac{\left({N}_{m}-{N}_{q}\right)}{{S}_{m}R} +{t}_{s}$$

which reduces to the FORM model in the case where all sites begin recruitment simultaneously.

#### Implementation: deterministic conditional

The deterministic conditional method was implemented for the only multicenter case-study RCT with site-specific expected recruitment rates available. At the design stage, recruitment predictions were calculated based on an anticipated recruitment rate of two participants per week at the chief investigator site, and one to two participants per week at other sites. This was compared to observed participant consent dates per site per month, which were assumed to approximate recruitment dates.

#### Unconditional stochastic poisson

Unlike deterministic models, stochastic models incorporate random fluctuations. The unconditional Poisson model, also referred to as a Homogenous Poisson Process (HPP), involves the occurrence of events one at a time, at a constant average rate, and independent of prior events. The average recruitment rate can be used to estimate the time to reach the target sample size via Poisson distribution simulations [15]. Let λ denote the expected number of participants recruited per day across all centres and let T denote the time in days to recruit N subjects, where N is equivalent to the required sample size. Let ${N}_{t}$ represent the total number of participants recruited before time t, with t<T, and let ${X}_{t}$ be the number of participants recruited on day t such that ${N}_{t+1}={N}_{t}+{X}_{t}$. Denote $P\left({N}_{t}=n\right)$ as the probability that n participants have been recruited before time t, then

6 $$P\left({X}_{t}=n\right)=\frac{{e}^{-\lambda t}{\left(\lambda t\right)}^{n}}{n!}$$

is a Poisson distribution with mean λt. A Normal distribution approximation, assuming a constant average recruitment rate over time for each site, can be used to estimate the time required to reach the target enrolment [15]. Alternatively, the model can be rearranged to estimate the required accrual rate for a fixed period. A limitation of the HPP model is that it does not account for varying start dates of recruitment between sites, nor varying recruitment rates across centres, nor variations in the number of centres over time.

#### Conditional stochastic poisson

The method of Carter [15] can be extended to produce time-varying recruitment predictions by specifying average accrual rates for each time interval which accommodates staggered site openings or seasonality, also referred to as a Non-Homogenous Poisson Process (NHPP).

The randomness in λ, the occurrence rate of events in the Poisson process, is introduced by denoting ${\lambda}_{kt}$ as the expected number of participants recruited at site *k* = 1,…,*K* on day *t*. Therefore, the total number of new participants recruited on day t is given as follows:

7 $$P\left({X}_{t}=x\right)=\frac{{e}^{-{\lambda}_{t}^{*}}{\left({\lambda}_{t}^{*}\right)}^{x}}{x!}$$

where ${\lambda}_{t}^{*}={\sum}_{K}{\lambda}_{kt}$ is the expected number of participants recruited on day t across all K sites. Simulations of random values of x can be carried out under this Poisson distribution until the target sample size is reached, enabling the calculation of percentiles for the recruitment time (T) from the empirical distribution. This approach avoids the assumption of a constant recruitment rate, beneficial in RCT planning.

#### Implementation: unconditional and conditional poisson

The ‘hpp.scenario’ function from the ‘poisson’ R package [16], discontinued with an archived version available, was used to obtain recruitment rate predictions for all case-study RCTs at the design stage using the unconditional and conditional Poisson methods, through the simulation of Poisson processes. An HPP and an NHPP were implemented, with the following parameters prespecified: target sample size, trial duration, and lambda (*λ*)—the occurrence rate of events in the Poisson process—calculated as the target sample size divided by the expected duration. 1000 simulations were conducted to balance accuracy and computational efficiency, and 100 points were used to estimate the mean and quantiles, as recommended by the ‘poisson’ package documentation. The additional inclusion of non-homogenous recruitment time for the NHPP model enabled the modelling of a recruitment rate which begins low, increases to a peak and then stabilises. A limitation of this model is the selection of this timepoint, especially in single-centre trials, and so this method was only implemented for multicentre trials with varying start dates to reflect the gradual opening of centres.

#### Bayesian

Bayesian approaches can aid the monitoring of recruitment predictions during trial conduct using prior beliefs and ongoing recruitment data. However, here we focus solely on the formation of the prior which can be used to predict recruitment at the design stage. Gajewski et al. [17] developed the approach of Williford et al. [18] who established prior probabilities to inform the predictive distribution before the collection of any recruitment data. A model was introduced whereby average times between participants joining the study are assumed to be exponentially distributed with mean θ, and the prior distribution for θ is taken to be the inverse gamma distribution with parameters ${\mathrm{IG}}(nP, TP)$, later described as an informative prior. T represents the time the investigator supposes it will take to recruit to the target sample size (n) and P denotes their confidence that n can be reached by time *T*, with $P\in [\mathrm{0,1}]$, such that P=1 represents total confidence. In this case, the prior sample size is taken to be the target sample size, and otherwise is reduced, i.e. to one-tenth if P=0.1 is chosen. TP scales the response so that the expected time between participants entering the study is approximately T/n. The authors state that prior accrual rates should be reliable given that means and medians are mostly accurately specified by experts, though P may be less readily available. In this case historical data from a similar trial can be utilised with care, for example with weighting based on the size of the previous study.

##### Implementation: Bayesian

Bayesian methods based on work by Gajewski et al. [17] and Jiang et al. [19] were implemented using the ‘accrual’ package in R to predict the rate of recruitment based on weighted linear regression [20]. For single-centre trials, the ‘accrual.n.plot’ function produced a prediction of the number of participants that could be recruited in a fixed time period using an informative prior. Inputs included the target timeframe, the time at which to make the prediction (set equal to this value) and the number of recruited participants and time to date, which were both set to zero in this case. Values of *P* of 0.01, 0.1, 0.3, 0.5, 0.7 and 0.9 were prespecified, representing low to high confidence that the target sample size would be reached within the required timeframe, with *P* = 0.01 representing an uninformative prior. To obtain predictions of the time in months to reach the specified sample size, the ‘accrual.T.plot’ function was utilised with inputs including the target sample size, the number of participants at which to make the prediction (set equal to this value), the number of recruited participants and time to date (both set to zero), and the same values of *P*. For multicentre trials, the ‘accrual.plot.multicenter’ function predicted recruitment in a fixed timeframe using an informative prior. Inputs included those used in the ‘accrual.n.plot’ function for single-centre trials in addition to the number of sites, start dates and individual recruitment numbers to date. A prediction of the time taken to recruit a prespecified sample size was not obtainable using this package.

### Case-study RCTs

Data were obtained from six parallel-group RCTs conducted at the Sheffield Centre for Health and Related Research (University of Sheffield). Ethical approval was granted by the University of Sheffield Research Ethics Committee for secondary use of anonymised data, originally collected with participant consent by the Clinical Trials Research Unit. The six case-study RCTs were selected by excluding pilot studies and multicentre trials that lacked site-specific opening dates, and to ensure a diverse range of trial characteristics. Patterns of recruitment were identified, and the recruitment prediction methods applied. Three of the six RCTs recruited to their target sample size, one recruited to slightly below target, and two failed to recruit an adequate number of participants. Table 1 summarises the key characteristics of the case-study RCTs: endometrial scratch to increase live birth rates in women undergoing first-time in vitro fertilisation (Endometrial Scratch) [21], a preventative lifestyle intervention for older adults (Lifestyle Matters) [22], the impact of pelvic floor muscle training on sexual function of women with urinary incontinence and a comparison of electrical stimulation versus standard treatment (IPSU) [23], the Journeying through Dementia (JtD) psychosocial intervention versus usual care study [24], a randomised controlled trial and cost-effectiveness evaluation of ‘booster’ interventions to sustain increases in physical activity in middle-aged adults in deprived urban neighbourhoods (Booster) [25], and the Putting Life in Years’ (PLINY) telephone friendship groups research study [26].

**Table 1:** Summary of case-study RCTs

Trial name	Accrual target	Total number accrued	Target met (Y/N)	Total centres opened	Accrual start date	Accrual end date	Total months of accrual
Endometrial Scratch	1044	1048	Y	16	May 2016	Oct 2018	28
Lifestyle Matters	268	288	Y	2	Aug 2012	Apr 2013	9
IPSU	114	114	Y	1	Oct 2011	Apr 2015	41
JtD	486	480	N	13	Feb 2017	Sep 2018	20
Booster	600	282	N	1	Nov 2009	Nov 2011	25
PLINY	248	157	N	1	Jun 2012	Jan 2013	8

**Fig. 1:**
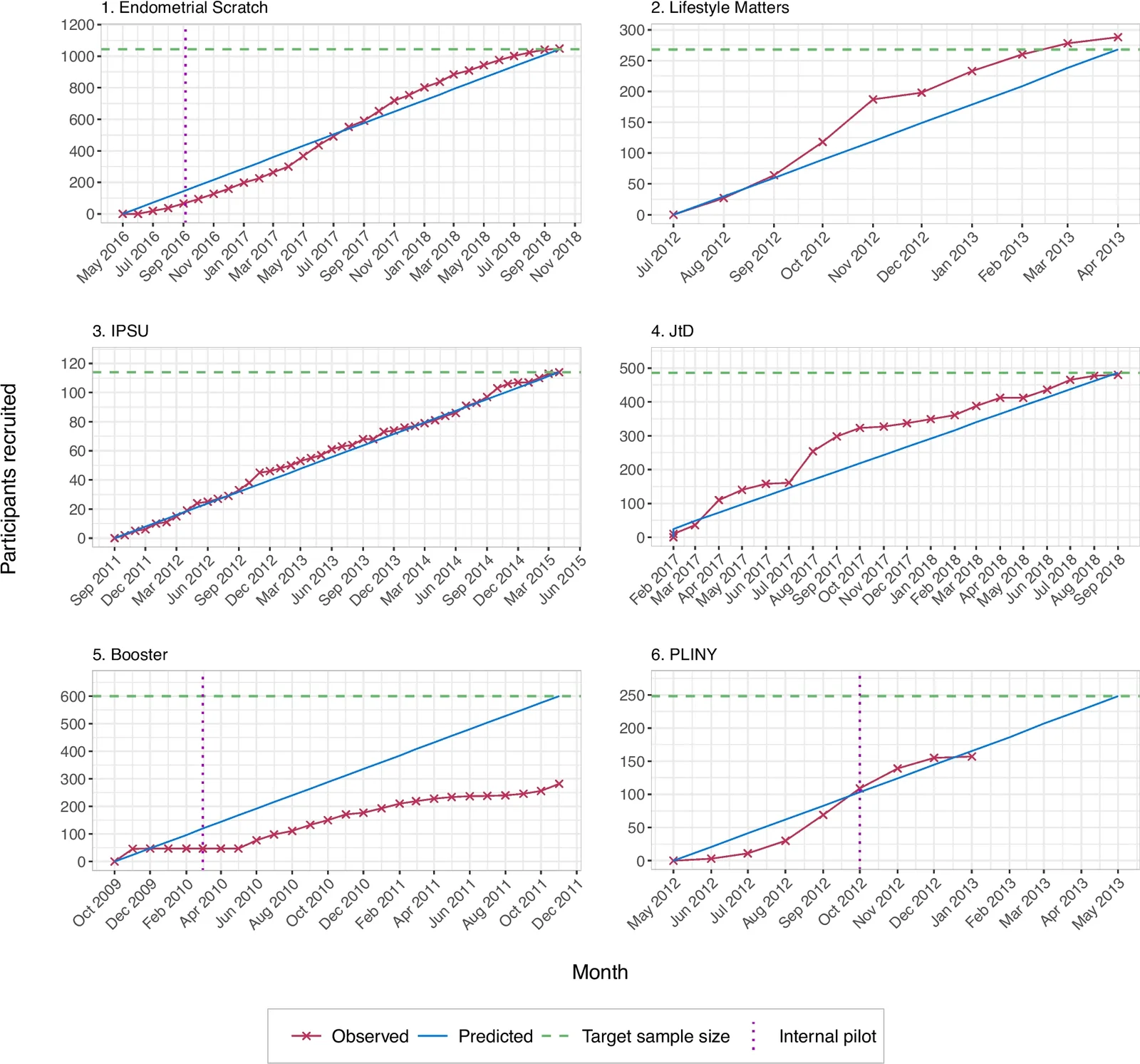
Case-study RCTs: observed recruitment compared to unconditional prediction

**Fig. 2:**
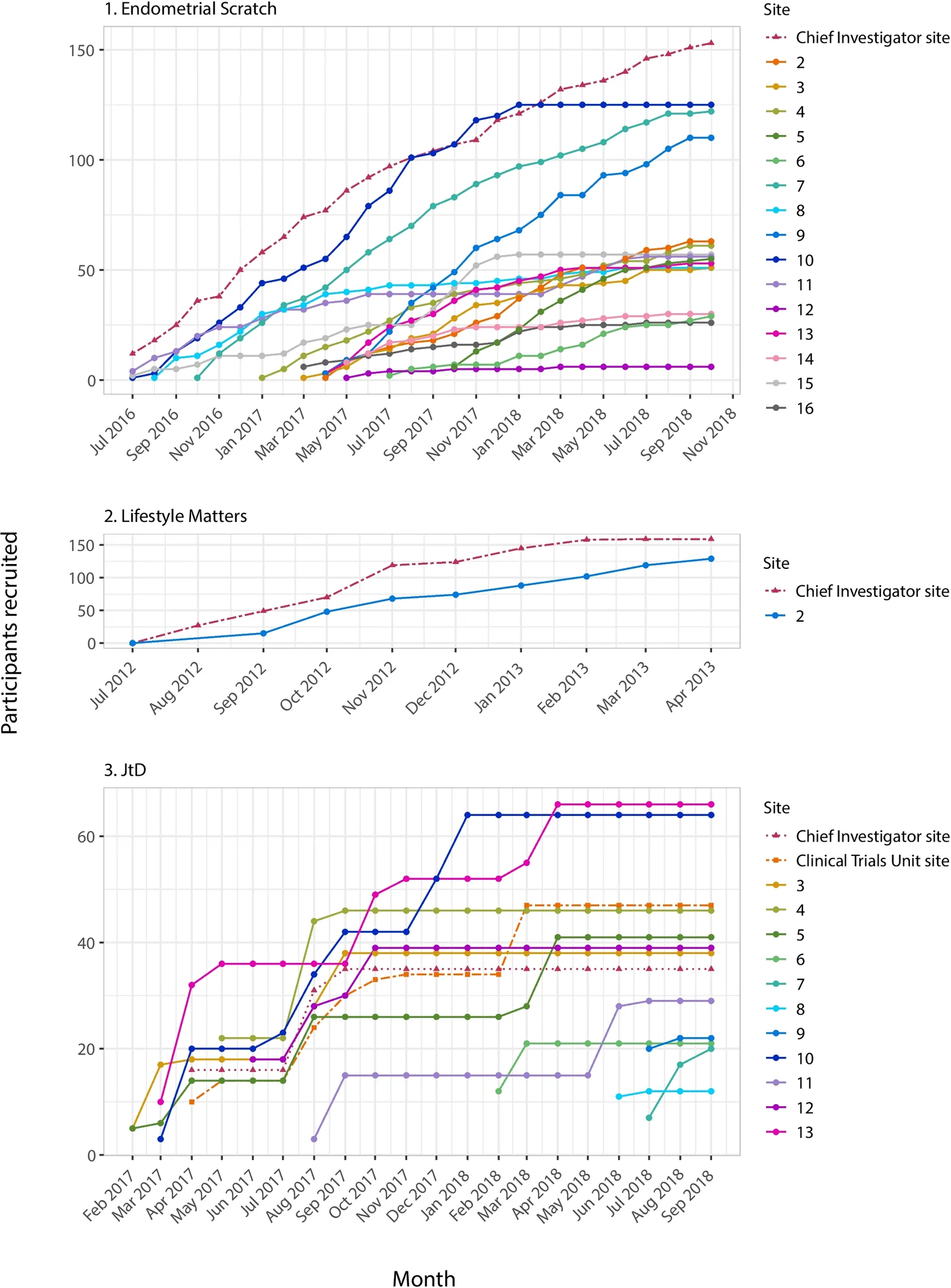
Multicentre case-study RCTs: observed recruitment by site

All trials continued recruitment until at least the protocol-defined timepoint, except for PLINY which closed recruitment 5 months prematurely due to a lack of recruitment and retention of volunteer facilitators resulting in a total recruitment period of 8 months. The Endometrial Scratch RCT used a conditional model for recruitment prediction, though for comparability an unconditional model was also applied. All other trials were assumed to have used an unconditional prediction method at the design stage as no statistical model was indicated. Figure 1 compares the observed recruitment with an unconditional prediction for each RCT and indicates those with internal pilot phases: Endometrial Scratch, Booster and PLINY. The unconditional prediction is simplistic, especially for multicentre trials, but is provided as a reference point for the comparison of other methods. Figure 2 presents observed recruitment by site for the three multicentre trials (Endometrial Scratch, Lifestyle Matters and JtD).

A commonality in recruitment across RCTs was a slower start, an increase to a maximum accrual rate midway through the trial, and a plateau at the end, regardless of whether the recruitment target was achieved. This trend was more pronounced in multicentre trials, often coinciding with the period where the most sites were active.

In terms of multicentre trials, Endometrial Scratch and Lifestyle Matters demonstrated higher recruitment rates at the chief investigator site. However, this trend was not observed for JtD, including at the Clinical Trials Unit site. Sites which started later tended to have slower accrual rates and recruit less participants overall.

Regarding trials with internal pilot phases, no consistent pattern emerged. Endometrial Scratch, which successfully reached its overall target, did not meet its internal pilot target. Booster failed to reach both its internal pilot and overall targets. In contrast, PLINY reached its internal pilot target but did not achieve its overall goal. This indicates that internal pilot success did not consistently predict overall recruitment outcomes.

Table 2 summarises the methods implemented and parameters used for each case-study RCT.

**Table 2:** Summary of methods implemented and parameters used

**Trial name**	**Deterministic unconditional** ***Target sample size; expected accrual time (months)***	**Deterministic conditional** ***Expected participants recruited per month/number of months at this rate (number of sites open)***	**Unconditional Poisson** ***Target sample size; expected accrual time (months)***	**Conditional Poisson** ***Target sample size; expected accrual time (months);non-homogenous recruitment time***	**Bayesian** ***Target sample size; expected accrual time (months); number of sites; month each site started recruiting***
Endometrial Scratch	1044; 28	10/7 (2); 30/2 (6); 50/19 (10)—based on anticipated recruitment rate of 2 per week at CI site, 1–2 per week at other sites	1044; 28	1044; 28; 15	1044; 28; 16; 1, 2, 2, 2, 2, 5, 8, 8, 8, 10, 10, 10, 10, 10, 12, 16
Lifestyle Matters	268; 9	NA	268; 9	268; 9; NA	268; 9; NA; NA
IPSU	114; 41	NA	114; 41	114; 41; NA	114; 41; NA; NA
JtD	486; 20	NA	486; 20	486; 20; 17	486; 20; 13; 1, 1, 2, 2, 3, 3, 4, 5, 7, 14, 17, 18, 18
Booster	600; 25	NA	600; 25	600; 25; NA	600; 25; NA; NA
PLINY	248; 13	NA	248; 13	248; 13; NA	248; 13; NA; NA

## Results

### Deterministic conditional

**Fig. 3:**
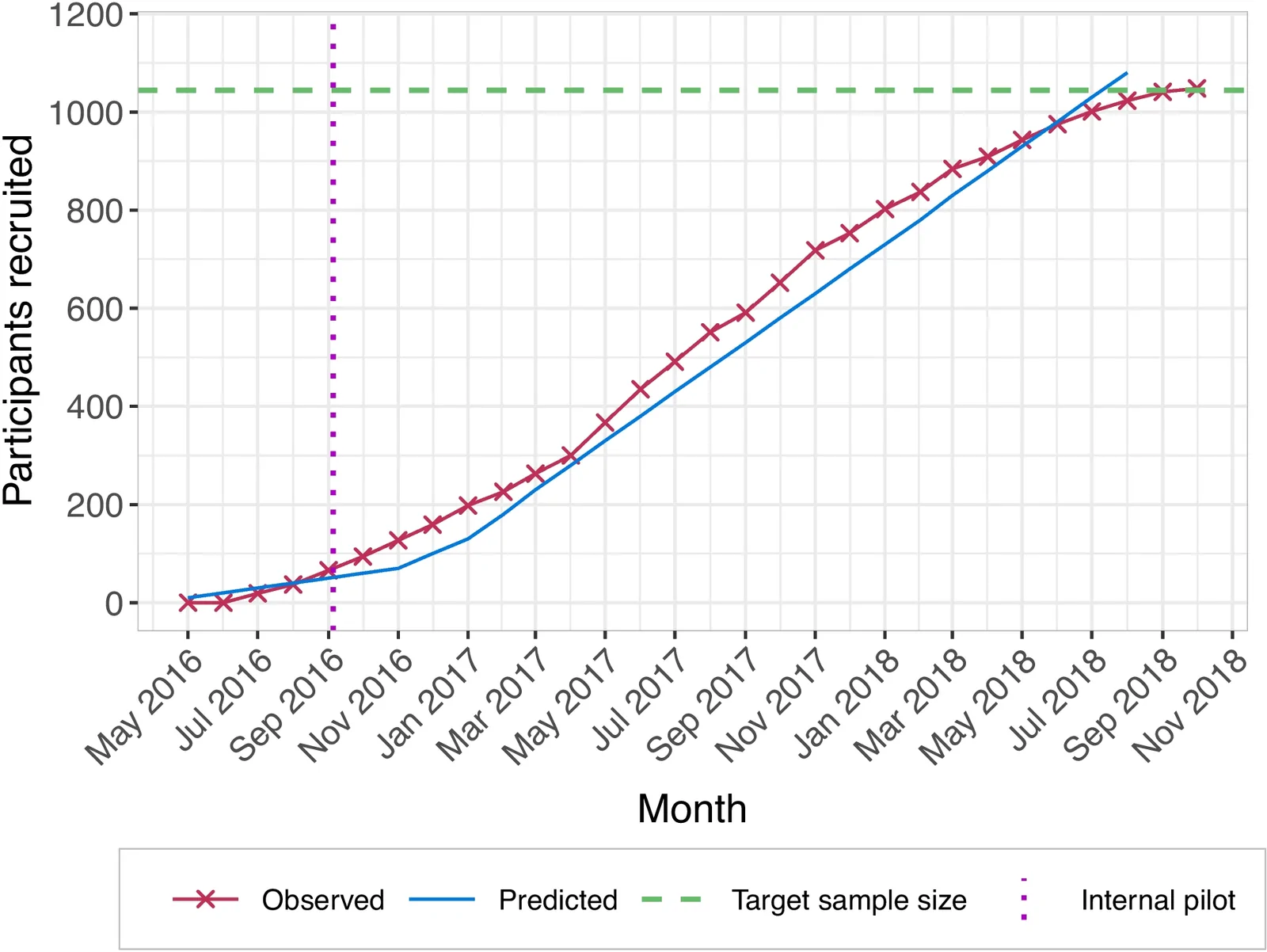
Endometrial Scratch RCT: observed recruitment compared to conditional prediction

Figure 3 presents the conditional model prediction compared to the observed accrual rate for Endometrial Scratch. Accrual was below the conditional prediction for the first 3 months, exceeded it until month 25, then plateaued, reaching the required 1044 participants 3 months after the target time of 28 months.

### Stochastic Poisson

Table 3 presents predictions of the time taken to recruit the respective target sample sizes and 95% confidence intervals using both a Homogenous and Non-homogeneous Poisson Process. The non-homogeneous recruitment timepoint was taken to be the first month at which all centres were open, months 15 and 17 for Endometrial Scratch and JtD respectively, after which the occurrence rate, *λ*, was assumed to follow the previously defined HPP occurrence rate.

**Table 3:** Predicted time to recruit target sample for Homogenous (HPP) and Non-Homogeneous Poisson (NHPP) process models

	**Recruitment target (months)**	***λ*ᵃ (participants per month)**	**HPP prediction**^**b**^** (95% confidence intervals, months)**	**NHPP prediction**^**c**^** (95% confidence intervals, months)**
Endometrial Scratch	28	37.3	(26.2, 29.8)	35.5 (33.8, 37.2)
Lifestyle Matters	9	29.8	(8.0, 10.1)	N/A
IPSU	41	2.8	(33.4, 48.6)	N/A
JtD	20	24.3	(18.2, 21.8)	28.5 (26.8, 30.3)
Booster	25	24	(23.1, 27.0)	N/A
PLINY	8	31	(7.0, 9.0)	N/A

ᵃThe occurrence rate of events in the Poisson process

^b^Prediction of the time taken to recruit the respective target sample size using a Homogenous Poisson Process (HPP); a point estimate cannot be obtained using this model

^c^Prediction of the time taken to recruit the respective target sample size using a Non-Homogenous Poisson Process (NHPP)

In general, the HPP offered more insight than deterministic approaches, providing quantiles which account for uncertainty, thereby approximating best and worst-case scenarios. However, the requirement of both a timeframe and a target sample size to calculate the expected recruitment rate hinders the obtainment of individual parameter predictions, as is possible with other methods. The confidence intervals for the HPP process were narrow, mostly with a width of around 3 months. This could help a funder assess the accuracy of the recruitment prediction. However, the availability of prespecified expected recruitment rates differs between trials. The use of a NHPP for multicentre trials gave more conservative predicted times to recruit to target, increasing the predictions by around 8 months in both cases compared to an HPP, though their accuracy may be greater. The widths of the confidence intervals were around 3 months, similar to those of the HPP predictions.

### Bayesian

**Fig. 4:**
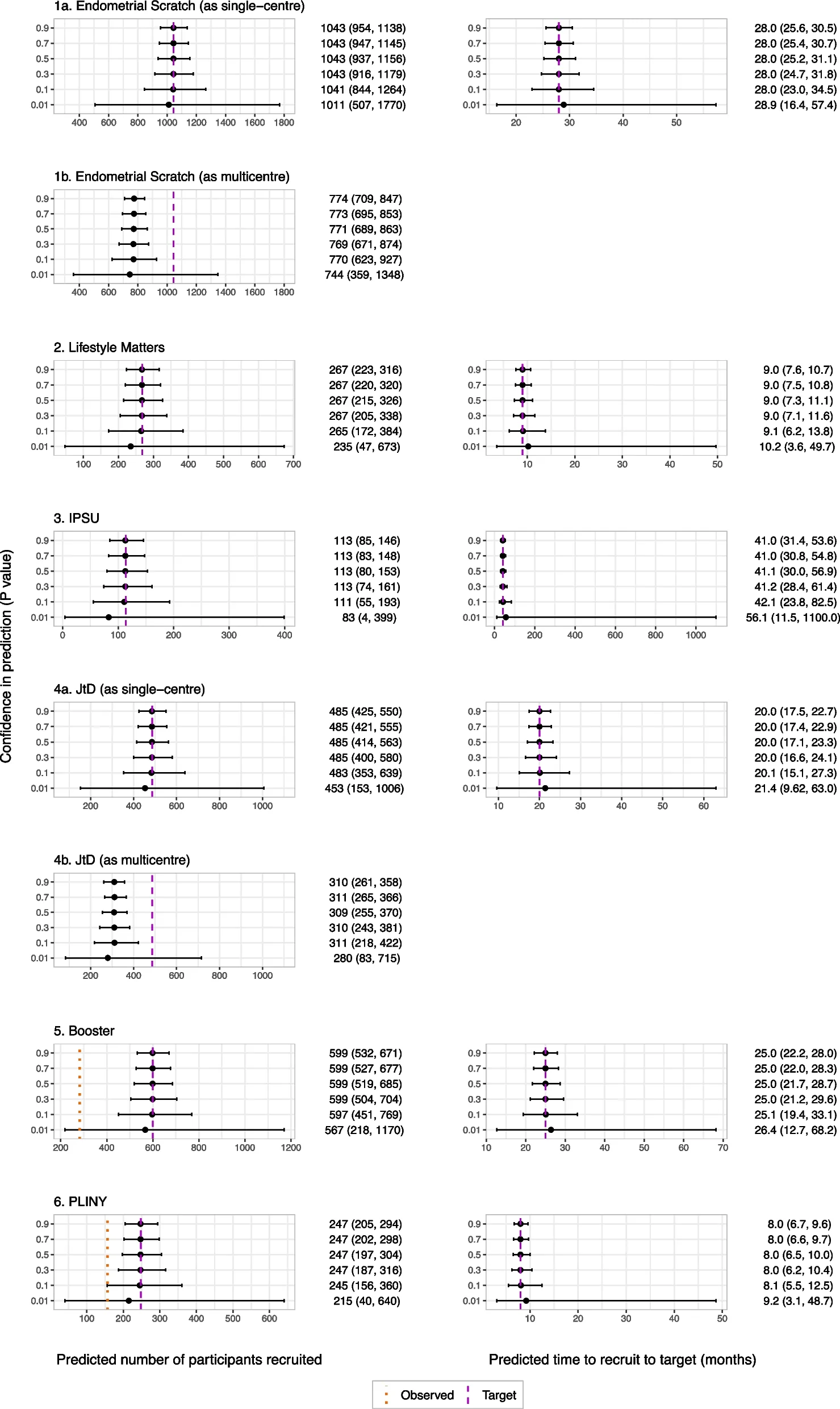
Predicted participants and recruitment time using Bayesian methods with informative prior and varying values of *P*

Figure 4 presents both predictions of the number of participants recruited in the specified timeframe and the time taken to recruit the target number of participants for all values of *P*. For the multicentre trials with varying start dates (Endometrial Scratch and JtD), predictions were obtained both accounting for the start dates and assuming all sites began recruitment simultaneously, as would be the case in a single-centre trial, in order to compare the impact of time-varying recruitment rates on the prediction method. As *P* increased, the credible intervals narrowed in all cases. The uninformative prior had a significantly wider width of around 500 participants depending on the size of the trial, reducing to a width of approximately 100–200 participants for values of *P* of 0.5 and above. Including site-specific start dates for the multicentre trials resulted in more conservative predictions, with smaller numbers of participants predicted. For RCTs which did not meet recruitment targets, Bayesian methods failed to predict the observed accrual at the design stage, though their accuracy may improve as the trials progress.

## Discussion

The suitability of methods for predicting both recruitment within a timeframe and time to reach a target sample size at the design stage depended on several factors: the prediction timepoint, whether the trial was single or multicentre, and whether the trial met its recruitment target. No clear association was found between the size of the RCT and the most suitable recruitment prediction method.

At the design stage, Poisson methods appeared to be the most appropriate, providing confidence intervals which could model uncertainty, thereby making them more useful to a funder than the single point estimates from deterministic unconditional and conditional methods. Additionally, Poisson intervals were narrower than the credible intervals produced by Bayesian methods. Despite these wider credible intervals, Bayesian methods offered relatively accurate point estimates based solely on prior information.

For single-centre trials, an HPP model was appropriate, whilst an NHPP model better addressed non-constant recruitment arising from staggered site start dates in multicentre trials. However, the NHPP model tended to yield more conservative estimates and relied on site-specific start dates, which may not be available at the design stage of an RCT. In any case, delayed site openings in multicentre trials should be carefully factored into recruitment predictions.

The limitations observed were consistent with those described by Carter [15] and Gajewski et al. [17]. These included challenges in parameter elicitation, such as predetermining accrual rates, and prespecifying prior beliefs along with their associated measures of certainty for Bayesian inference. Furthermore, facilitators in the implementation of these methods had barriers hindering their use, such as the discontinuation of the ‘poisson’ package [16], coding errors, and restricted functionality within the ‘accrual’ package [20]. Moreover, the methods were applied to data from six case-study trials which varied in characteristics. This provided a wider range of scenarios by encompassing trials which successfully and unsuccessfully recruited to the target sample size and varied in their duration and number of participants recruited. However, it impeded direct comparability between similar trials, which limited the ability to strengthen evidence for specific methods. Another limitation was a lack of information surrounding recruitment-enhancing measures, both trial-specific and generally, such as the introduction of the SEAR framework [27]. These strategies could have influenced the predictive accuracy of the models or impacted the reproducibility of results, depending on how they were applied.

It should also be noted that there exist more advanced techniques for predicting recruitment in multicentre trials at the design stage [28, 29]. In particular, the Poisson-Gamma model can account for random variation of rates across centres, the number of centres over time and centre initiation dates. This model could not be implemented using these case-study trials due to a lack of historical data from similar trials, clinical information or expert estimators. Therefore, this is not a comprehensive list of methods but rather a demonstration of how some methods may be practically applied at the design stage of a trial. Readers are encouraged to explore these models if they have access to more information prior to starting recruitment, or if they are looking to update recruitment predictions as the trial progresses.

## Conclusions

Recruitment to the target sample size in RCTs is a well-recognised, frequent issue, making the prediction and monitoring of recruitment during the conduct of an RCT critical. This research aimed to evaluate the most appropriate statistical method for predicting recruitment at the design stage of a trial. Poisson methods emerged as the more appropriate approach, although additional research is required to further substantiate these findings.

It is recommended that RCTs publish the methods and parameters used in their recruitment predictions to enable further research and facilitate method comparisons across a broader range of trials. For instance, although 99% of 125 RCTs from five major journals reported a target sample size, only 15% reported the expected recruitment period [30]. This could impede the implementation of Poisson methods which require a prespecified recruitment rate and offer a balance between ease of implementation, prediction accuracy and incorporation of uncertainty. Improved reporting of recruitment rates, including site-specific rates, could better inform these parameters and thereby facilitate the implementation of these methods. Further research into Bayesian methods should also be conducted to formalise the elicitation procedure and streamline their implementation.

To the best of our knowledge, no prior comparison of these methods using data from RCTs has been carried out previously. Further research using a wider range of case-studies and methods would be recommended to further ascertain the most appropriate statistical method for predicting recruitment in randomised controlled trials.

## Data Availability

The datasets used and/or analysed during the current study are available from the corresponding author on reasonable request.

## Abbreviations

RCT: Randomised Controlled Trial
HPP: Homogenous Poisson Process
NHPP: Non-homogeneous Poisson Process

## References

[1] Kasenda B, von Elm E, You J, et al. Prevalence, characteristics, and publication of discontinued randomized trials. JAMA. 2014;311:1045–51. 10.1001/jama.2014.1361 24618966

[2] Vellinga A, Lambe K, O’Connor P, O’Dea A. What discontinued trials teach us about trial registration? BMC Res Notes. 2021;14:47. 10.1186/s13104-020-05391-w PMC7863519 33546746

[3] NIH (2023) National Institutes of Health (NIH) Funding: FY1996-FY2023. CRS Rep R43341

[4] NIHR (2024) NIHR Annual Report 2022/23. https://www.nihr.ac.uk/reports/nihr-annual-report-202223/34501. Accessed 14 Jun 2024

[5] Jacques RM, Ahmed R, Harper J, Ranjan A, Saeed I, Simpson RM, et al. Recruitment, consent and retention of participants in randomised controlled trials: a review of trials published in the National Institute for Health Research (NIHR) Journals Library (1997–2020). BMJ Open. 1997;12:1–11. 10.1136/bmjopen-2021-059230 PMC8845327 35165116

[6] McDonald AM, Knight RC, Campbell MK, et al. What influences recruitment to randomised controlled trials? A review of trials funded by two UK funding agencies. Trials. 2006;7:9. 10.1186/1745-6215-7-9 PMC1475627 16603070

[7] Walters SJ, Dos Anjos Henriques-Cadby IB, Bortolami O, et al. Recruitment and retention of participants in randomised controlled trials: a review of trials funded and published by the United Kingdom Health Technology Assessment Programme. BMJ Open. 2017. 10.1136/bmjopen-2016-015276. PMC5372123 28320800

[8] Toerien M, Brookes ST, Metcalfe C, de Salis I, Tomlin Z, Peters TJ, et al. A review of reporting of participant recruitment and retention in RCTs in six major journals. Trials. 2009. 10.1186/1745-6215-10-52. PMC2717957 19591685

[9] Moffat KR, Shi W, Cannon P, Sullivan F. Factors associated with recruitment to randomised controlled trials in general practice: a systematic mixed studies review. Trials. 2023;24:90. 10.1186/s13063-022-06865-x PMC9903494 36747260

[10] Gkioni E, Dodd S, Rius R, Gamble C. Statistical models to predict recruitment in clinical trials were rarely used by statisticians in UK and European networks. J Clin Epidemiol. 2020;124:58–68. 10.1016/j.jclinepi.2020.03.012 32229249

[11] Barnard KD, Dent L, Cook A. A systematic review of models to predict recruitment to multicentre clinical trials. BMC Med Res Methodol. 2010. 10.1186/1471-2288-10-63. PMC2908107 20604946

[12] Gkioni E, Rius R, Dodd S, Gamble C. A systematic review describes models for recruitment prediction at the design stage of a clinical trial. J Clin Epidemiol. 2019;115:141–9. 10.1016/j.jclinepi.2019.07.002 31299358

[13] Carter RE, Sonne SC, Brady KT. Practical considerations for estimating clinical trial accrual periods: application to a multi-center effectiveness study. BMC Med Res Methodol. 2005;5:1–5. 10.1186/1471-2288-5-11 PMC1079860 15796782

[14] Comfort S (2013) Improving clinical trial enrollment forecasts using SORM Comfort, Shaun, MD.Applied Clinical Trials; Monmouth Junction Vol. 22, Iss. 5, (May 2013): 32,34–38.

[15] Carter RE. Application of stochastic processes to participant recruitment in clinical trials. Control Clin Trials. 2004;25:429–36. 10.1016/j.cct.2004.07.002 15465613

[16] Brock K, Slade D (2015) Package ‘poisson’: simulating Homogenous & NonHomogenous Poisson Processes. R package version 1.0.

[17] Gajewski BJ, Simon SD, Carlson SE. Predicting accrual in clinical trials with Bayesian posterior predictive distributions. Stat Med. 2008;27:2328–40. 10.1002/sim.3128 17979152

[18] Williford WO, Bingham SF, Weiss DG, Collins JF, Rains KT, Krol WF. The “constant intake rate” assumption in interim recruitment goal methodology for multicenter clinical trials. J Chronic Dis. 1987;40:297–307. 10.1016/0021-9681(87)90045-2 3558706

[19] Jiang Y, Simon S, Mayo MS, Gajewski BJ. Modeling and validating Bayesian accrual models on clinical data and simulations using adaptive priors. Stat Med. 2015;34:613–29. 10.1002/sim.6359 PMC4314351 25376910

[20] Liu, Y J, C W, S S, MS M, Ra R (2023) Bayesian accrual prediction. R package version 1.3. 10.32614/CRAN.package.accrual

[21] Metwally M, Chatters R, Pye C, et al. Endometrial scratch to increase live birth rates in women undergoing first-time in vitro fertilisation: RCT and systematic review. Health Technol Assess (Rockv). 2022;26:vii–122. 10.3310/JNZT9406 PMC8859770 35129113

[22] Mountain G, Windle G, Hind D, et al. A preventative lifestyle intervention for older adults (lifestyle matters): a randomised controlled trial. Age Ageing. 2017;46:627–34. 10.1093/ageing/afx021 PMC5860501 28338849

[23] Jha S, Walters SJ, Bortolami O, Dixon S, Alshreef A. Impact of pelvic floor muscle training on sexual function of women with urinary incontinence and a comparison of electrical stimulation versus standard treatment (IPSU trial): a randomised controlled trial. Physiother (United Kingdom). 2018;104:91–7. 10.1016/j.physio.2017.06.003 28801034

[24] Mountain GA, Cooper CL, Wright J, et al. The Journeying through Dementia psychosocial intervention versus usual care study: a single-blind, parallel group, phase 3 trial. Lancet Heal Longev. 2022;3:e276–85. 10.1016/S2666-7568(22)00059-9 36098301

[25] Goyder E, Hind D, Breckon J, et al. A randomised controlled trial and cost-effectiveness evaluation of “booster” interventions to sustain increases in physical activity in middle-aged adults in deprived urban neighbourhoods. Health Technol Assess (Rockv). 2014;18:1–209. 10.3310/hta18130 PMC4781193 24571932

[26] Hind D, Mountain G, Gossage-Worrall R, et al. Putting life in years (PLINY): a randomised controlled trial and mixed-methods process evaluation of a telephone friendship intervention to improve mental well-being in independently living older people. Public Health Res. 2014;2:1–222. 25642518

[27] Wilson C, Rooshenas L, Paramasivan S, et al. Development of a framework to improve the process of recruitment to randomised controlled trials (RCTs): the SEAR (Screened, eligible, approached, randomised) framework. Trials. 2018;19:1–10. 10.1186/s13063-017-2413-6 PMC5775609 29351790

[28] Austin M, Anisimov V. Forecasting and optimizing patient enrolment in clinical trials under various restrictions. Springer Proc Math Stat. 2022. 10.1007/978-3-031-17820-7_23.

[29] Anisimov VV. Statistical modeling of clinical trials (recruitment and randomization). Commun Stat - Theory Methods. 2011;40:3684–99.

[30] Gkioni E (2021) Improving the planning and monitoring of recruitment to clinical trials.

